# Diabetes mellitus increased integrins gene expression in rat endometrium at the time of embryo implantation

**DOI:** 10.18502/ijrm.v17i6.4810

**Published:** 2019-07-29

**Authors:** Abbas Bakhteyari1 Ph.D. candidate, Yasaman Zarrin, Parvaneh Nikpour, Zeinab Sadat Hosseiny, Fatemah Sadat Mostafavi, Nahid Eskandari, Mohammad Matinfar, Roshanak Aboutorabi

**Affiliations:** ^1^Department of Anatomical Sciences, Faculty of Medicine, Isfahan University of Medical Sciences, Isfahan, Iran.; ^2^Department of Genetics and Molecular Biology, Faculty of Medicine, Isfahan University of Medical Sciences, Isfahan, Iran.; ^3^Child Growth and Development Research Center, Research Institute for Primordial Prevention of Non-communicable Disease, Isfahan University of Medical Sciences, Isfahan, Iran.; ^4^Department of Immunology, Faculty of Medicine, Isfahan University of Medical Sciences, Isfahan, Iran.; ^5^Department of Internal Medicine Faculty of Medicine, Isfahan University of Medical Sciences, Isfahan, Iran.

**Keywords:** Diabetes mellitus, Embryo implantation, Integrins, Endometrium.

## Abstract

**Background:**

Diabetes mellitus deeply changes the genes expression of integrin (*Itg*) subunits in several cells and tissues such as monocytes, arterial endothelium, kidney glomerular cells, retina. Furthermore, hyperglycemia could impress and reduce the rate of successful assisted as well as non-assisted pregnancy. Endometrium undergoes thorough changes in normal menstrual cycle and the question is: What happens in the endometrium under diabetic condition?

**Objective:**

The aim of the current study was to investigate the endometrial gene expression of α3, α4, αv, *Itg* β1 and β3 subunits in diabetic rat models at the time of embryo implantation.

**Materials and Methods:**

Twenty-eight rats were randomly divided into 4 groups: control group, diabetic group, pioglitazone-treated group, and metformin-treated group. Real-time PCR was performed to determine changes in the expression of *Itg* α3, α4, αv, β1, and β3 genes in rat's endometrium.

**Results:**

The expression of all *Itg* subunits increased significantly in diabetic rats' endometrium compared with control group. Treatment with pioglitazone significantly reduced the level of *Itg* subunits gene expression compared with diabetic rats. While metformin had a different effect on α3 and α4 and elevated these two subunits gene expression.

**Conclusion:**

Diabetes mellitus significantly increased the expression of studied *Itg* subunits, therefore untreated diabetes could be potentially assumed as one of the preliminary elements in embryo implantation failure.

## 1. Introduction

Diabetes mellitus (DM) is referred to a member of metabolic disorders whose common feature is high blood glucose level. Diabetic individuals are at more life-threatening risk than normoglycemic people which can lead to increase in their healthcare costs and reduce their quality of life (1). The dramatic increase in the number of people with diabetes is due to population aging, changes in lifestyle, obesity, and population growth (2). Type 2 diabetes mellitus (T2DM) or noninsulin-dependent diabetes mellitus (NIDDM) is diagnosed in about 90% of cases before age 30. It ought to be considered, this age is approximately in the middle range of women's fertility age (2). Platt and colleagues have reported that the rate of abortion after the implantation of blastocyst in diabetic women is nine times more than non-diabetic and healthy women (3).

The World Health Organization has reported that 8–10% of couples in the world are suffering from infertility, which is considered as a medical problem (2). The specific mechanisms of the preliminary causes of pregnancy failure, spontaneous and frequent abortions in diabetic women have not yet been clearly identified (4).

Unexplained infertility has several potential causes, one of which may be embryo implantation failure (5). It seems that these events could probably be in relation to underlying metabolic disorders such as DM (6). In humans and animals, the embryo implantation and the onset of pregnancy requires the occurrence of temporary and extremely planned events which provide and equip the endometrium at a specific time for blastocyst acceptance. This specific time is termed as the “window of implantation” (4, 7). During this period of time, blastocyst binds to the maternal tissue and adheres to endometrial epithelium, triggering a cascade of complex events that ultimately leads to the development of the placenta and embryo (8).

Genes and proteins that are involved in several uterine mechanisms, including cytokines/chemokines, growth factors, enzymes, aquaporins, and cell adhesion molecules such as cadherins, selectins, and integrins (*Itg*), play roles in regulating embryo-uterine dialogue at the time of implantation (9).

Several *Itg* subunits including α3, α4, αv, β1, and β3 have been identified in luminal and glandular endometrial epithelium (5). α3β1 *Itg* has a high tendency to bind with collagen, laminin, and fibronectin in extracellular matrix. But α4β1 *Itg* is known as a specific receptor for laminin, and the αvβ3 *Itg* is a major fibronectin receptor (10, 11). Diabetes and subsequent increases in blood glucose have been reported to affect the expression of *Itg* (12). In diabetic nephropathy rat model, αv, β1, and β3 *Itg* subunits gene expression was significantly upregulated compared to the control group in the renal cortex (13). In the early stage of diabetic nephropathy in human, α3β1 *Itg* gene expression significantly rose (14).

During a normal and natural menstrual cycle, the *Itg* α4, αv, β1, and β3 subunits should be periodically expressed in the endometrium at the exact site and appropriate amount (15). In cases with implantation failures, down regulation of αvβ3 expression were observed in endometrium (5, 16). In mice and rabbits, blockage of *Itg* αv and β3 subunits could lead to reduction in the number of implantation sites (17) and implantation failure could occur at the time of endometrial penetration in β1 subunit null mice (18). As mentioned earlier, DM could impress on *Itg* subunits gene expression in different tissues and organs (12, 19).

Metformin is considered as the first line for treatment in DM, if there is no contraindication (20). Pioglitazone is one of the subsets of thiazolidinedione (TZDs) family which is widely used for the treatment of DM. This drug has attracted the attention due to its multiple impressions beyond controlling hyperglycemia in liver, muscle, and adipose tissue by cell insulin-sensitizing activity and its effect on peroxisome proliferator-activated receptor-gamma (PPARγ) (21).

The aim of the current study was to investigate the endometrial gene expression of *Itg* α3, α4, αv, β1, and β3 subunits in diabetic rat model at the time of embryo implantation. We furthermore assessed the effects of metformin and pioglitazone treatment on the expression level of these *Itg* subunits in the endometrium tissue of diabetic rats.

## 2. Materials and Methods

### Animals

This interventional experimental study was performed on diabetic rat models at the central laboratory of Isfahan University of Medical Sciences in 2018. Adult virgin female Wistar rats weighting 200 ± 25 gr, aged around six weeks were purchased from the Pasteur Institute of Iran. Animals were maintained in constant humidity (40-70%), air-conditioned quarters (temperature 22-24°C) and 12 hr light/dark photoperiod (22).

### Study design and sampling

Twenty eight rats were randomly divided into four groups with seven rats each as follows: Control group, STZ + NA-induced diabetic group without any treatment (FBS ≥ 250 mg/dl), diabetic group that received Pioglitazone 20 mg/kg/day by orogastric gavage, and diabetic rats that received metformin 100 mg/kg/day by orogastric gavage.

### Induction of diabetes

In order to induce experimental T2DM in animals, Nicotinamide (NA, Sigma-Aldrich, Germany) and Streptozotocin (STZ, Sigma-Aldrich, Germany) were used. First, Nicotinamide (200-230 mg/kg) and 15 min later, 60 mg/kg Streptozotocin were intraperitoneally injected (23). After three days, blood samples were taken to measure fasting blood sugar (FBS) using a glucometer (HemoCue Glucose 201+, Sweden). Animals with a blood glucose level above 250 mg/dl were considered as a diabetic model. Animals were maintained in diabetic condition for four weeks. Then during the next four weeks, they received hypoglycemic drugs. FBS levels were measured every four days using glucometer (HemoCue Glucose 201+, Sweden) and glucose reagent strips (ACCU-CHEK Active, Germany) through the dorsal vein of rats' tail. Twenty four days after the administration of metformin or pioglitazone, two female and one male rats were placed in one cage in all study groups in order to mate animals. The day after that, the rats' vagina was checked. The presence of the vaginal plug revealed the first day of pregnancy and the time of implantation window was considered four days later (28${}^{\mathrm{th}}$ day after the beginning of the treatment by both drugs). Four weeks after the treatment with metformin or pioglitazone, at the time of implantation, animals fasted overnight and were then sacrificed by intraperitoneal injection of Ketamine hydrochloride (50 mg/kg) and Xylazine hydrochloride (7 mg/kg). Uterine horns were dissected and removed in a sterile condition. Uteri were washed with Hanks' balanced salt solution and chopped in several fragments. Tissues were then snap-frozen and kept in -80°C until performing further experiments.

### Total RNA isolation and cDNA synthesis

Total RNA was extracted from ‎‏endometrium tissue using RNX-plus solution (Sinaclon, Iran) as stated in manufacturer's protocol. To determine RNA integrity, 1% agarose gel electrophoresis was used and total RNA concentrations were evaluated by Nanodrop instrument (Nanolytik, Germany) at an optical density of 260 nm and stored at -80°C for the next steps. Using DNase set (Fermentas, Lithuania), DNase I treatment was performed in order to eliminate genomic DNA in the RNA samples, Synthesis of cDNA was performed from 1 µg of total RNA, by means of PrimeScriptTM RT reagent Kit (TaKaRa, Japan) as stated in the protocol.

### Quantitative real-time polymerase chain reaction

The expression level of *Itg* α3, α4, αv, β1, and β3 genes were assessed by quantitative real-time reverse transcription-polymerase chain reaction (RT-PCR). Specific primers (Table I) were designed utilizing GeneRunner software, version 4.0 (Hastings Software, Inc.) and tested by BLAST (Basic Local Alignment Search Tool (http://blast.ncbi.nlm.nih.gov/Blast.cgi)) for specific attachment to the genome. The β-actin gene was considered as the housekeeping gene (24). Real-time PCR was carried out with RealQ Plus ×2 Master Mix, green (high ROX) (AMPLIQON, Denmark) on an Applied Biosystems StepOnePlus${}^{TM}$ instrument. Standard cycling steps were used to perform Real-time PCR. Amplification conditions included of the first denaturation at 95°C for 10 min, 40 cycles of denaturation at 95°C for 15 sec, annealing at a specific temperature for each gene, according to Table I, for 60 sec, then an extension for 15 sec at 72°C.

**Table 1:** Primer sequences used in the Real-time PCR technique


**Primer name**	**Sequence**	**Tm (°C)**	**Annealing temperature (°C)**	**Amplicon size (bp)**
β-actin-F	5´-GCCTTCCTTCCTGGGTATG-3´	63.4	
β-actin-R	5´-AGGAGCCAGGGCAGTAATC-3´	63	60	178
*Itg* α3-F	5´-AGCAGCCTCAGCAGATAATC-3´	61.2			
*Itg* α3-R	5´-GGAGGATATTGATGACAGGTC-3´	59.8	56.8	176
*Itg* α4-F	5´-GCATACGAGTTTCAAGCAG-3´	57.7			
*Itg* α4-R	5´-CTCTGTGTTTCGTTTGGTG-3´	57.4	54.4	142
*Itg* αv-F	5´-TGTCAAGGAGGATTCAGC-3´	56.1			
*Itg* αv -R	5´-AGTCCGTGTGGCTAGTTG-3´	56.6	53.1	174
*Itg* β1-F	5´-TACTTCAGACTTCCGCATTG-3´	61.3			
*Itg* β1-R	5´-GCTGCTGACCAACAAGTTC-3´	59.4	56.4	191
*Itg* β3 -F	5´-GTCGTCAGCCTTTACCAG-3´	56.5			
*Itg* β3- R	5´-GAGATCACGCACTTCCAG-3´	56.3	53.3	164

### Ethical consideration

The Isfahan University of Medical Sciences Institutional Animal Ethical Committee approved all the experimental procedures (IR.MUI.REC.1394.1.184).

### Statistical analysis

In order to quantify the relative values of gene expression, 2-Δ ΔCT method (25) was used. Statistical analysis was performed using Statistical Package for the Social Sciences (SPSS) software, version 16.0. All experiments were performed at least twice or thrice and final results were expressed as means ± standard deviation (SD). One-Sample Kolmogorov–Smirnov Test was performed in order to evaluate normality. One-way ANOVA and Bonferroni post-test were performed to detect statistical significance which was considered as p < 0.05.

## 3. Results

### The gene expression of α3 integrin

**Figure 1:**
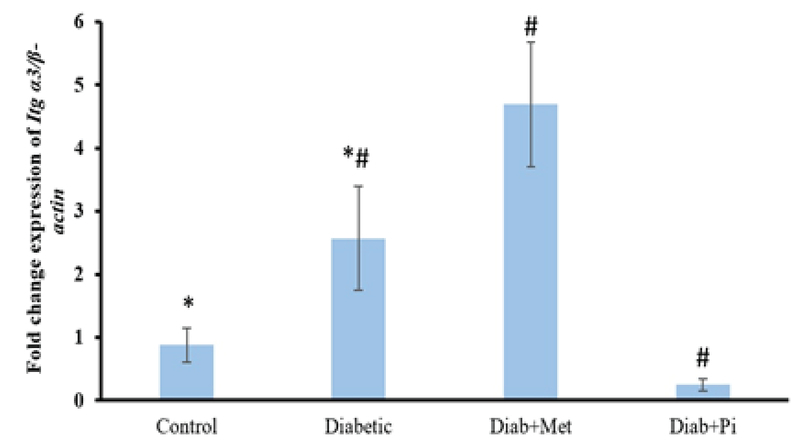
Comparison of *Itg* α3 gene expression at the time of embryo implantation in the rat endometrium. All values were presented as mean ± SD; p < 0.05 was considered statistically significant; Diab + met = diabetic group treated with metformin and Diab + Pi = diabetic group treated with piglitazone; * shows significant difference between diabetic group and control group; # shows significant difference between diabetic group compared with Diab + met group and Diab + Pi group.

The expression of *Itg* α3 was significantly increased in diabetic group in comparison with control group (p < 0.001, Figure 1). Following the treatment by metformin, α3 gene expression was significantly raised. A significant decrease was observed after pioglitazone treatment compared with the diabetic group (p < 0.001).

### The gene expression of α4 integrin

**Figure 2:**
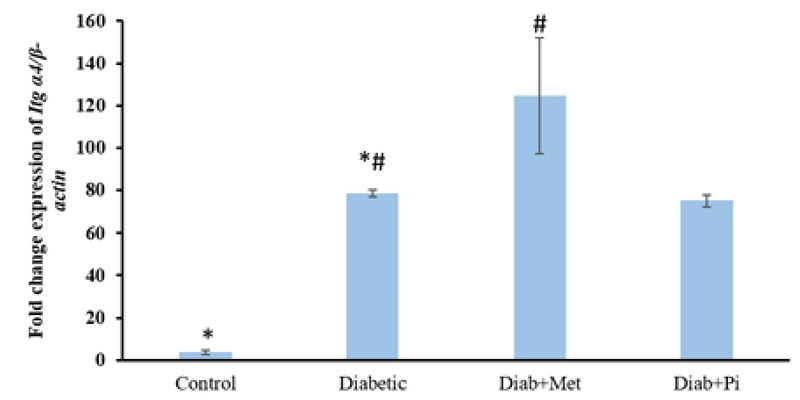
Comparison of *Itg* α4 gene expression at the time of embryo implantation in the rat endometrium. All values were presented as mean ± SD; p < 0.05 was considered statistically significant; Diab + met = diabetic group treated with metformin and Diab + Pi = diabetic group treated with piglitazone. * shows significant difference between diabetic group and control group; # shows significant difference between diabetic group compared with Diab + met group and Diab + Pi group.

As Figure 2 shows, the *Itg* α4 gene expression in the diabetic group was significantly higher than in the control group (p < 0.001). The level of *Itg* α4 gene expression in the pioglitazone-treated

group was lower than the diabetic group; however, this difference was not significant (p = 1.00). On the contrary, the level of *Itg* α4 gene expression in metformin-treated group was higher than the diabetic group, and a significant difference was observed in comparison with the diabetic group (p < 0.002).

### The gene expression of αv integrin

**Figure 3:**
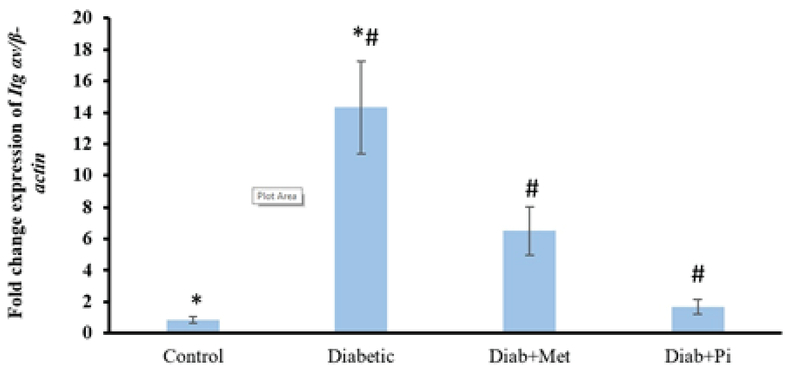
Comparison of *Itg* αv gene expression at the time of embryo implantation in the rat endometrium. All values were presented as mean ± SD; p < 0.05 was considered statistically significant; Diab + met = diabetic group treated with metformin and Diab + Pi = diabetic group treated with piglitazone. * shows significant difference between diabetic group and control group. # shows significant difference between diabetic group compared with Diab + met group and Diab + Pi group.

As Figure 3 illustrates, the results of the expression of *Itg* αv gene show that the expression in the diabetic group is higher than the other groups and is statistically significant in comparison with the control group (p < 0.001), metformin-treated group (p < 0.001), and pioglitazone-treated group (p < 0.001). Both the metformin and pioglitazone decreased the amount of αv gene expression compared with the diabetic group (p < 0.001).

### The gene expression of β1 integrin

**Figure 4:**
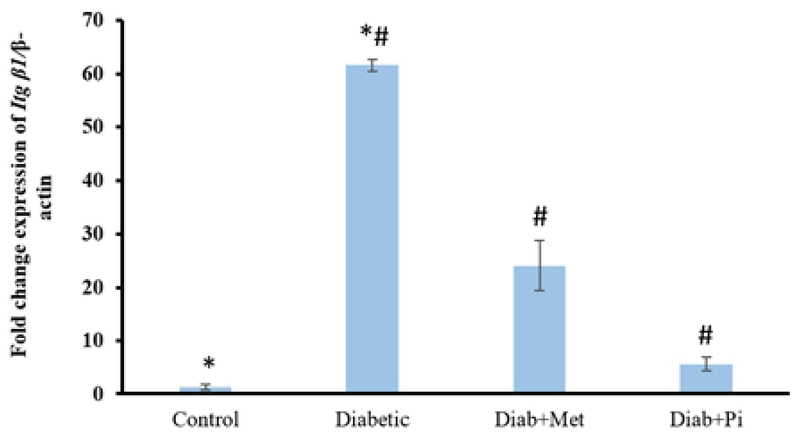
Comparison of *Itg* β1 gene expression at the time of embryo implantation in the rat endometrium. All values were presented as mean ± SD; p < 0.05 was considered statistically significant; Diab + met = diabetic group treated with metformin and Diab + Pi = diabetic group treated with piglitazone. * shows significant difference between diabetic group and control group; # shows significant difference between diabetic group compared with Diab + met group and Diab + Pi group.

As Figure 4 reveals, the mean of *Itg* β1 gene expression in diabetic group has significant difference in comparison with the control group (p < 0.001), metformin-treated group (p < 0.001), and pioglitazone-treated group (p < 0.001). After the treatment with both metformin and pioglitazone, the level of *Itg* β1 gene expression significantly decreased (p < 0.001).

### The gene expression of β3 integrin

**Figure 5:**
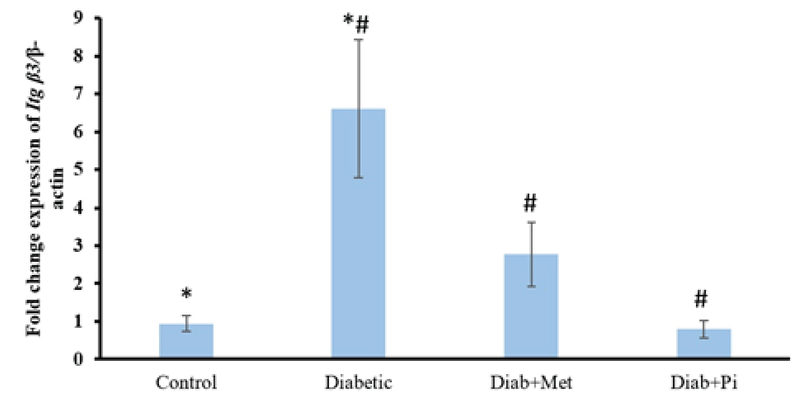
Comparison of *Itg* β3 gene expression at the time of embryo implantation in the rat endometrium. All values were presented as mean ± SD; p < 0.05 was considered statistically significant; Diab + met = diabetic group treated with metformin and Diab + Pi = diabetic group treated with piglitazone. * shows significant difference between diabetic group and control group; # shows significant difference between diabetic group compared with Diab + met group and Diab + Pi group.

Figure 5 demonstrates that its expression in the diabetic group has significant difference with the control group (p < 0.001) and significantly decreased after the treatment with metformin (p = 0.001) and pioglitazone (p < 0.001).

## 4. Discussion

DM is swiftly going to become one of the serious health problems around the world affecting several tissues and organs (2). The association of DM with *Itg* subunits genes and proteins expression changes has already been proven in various tissues and organs such as kidney, retina, arterial endothelium, and astrocytes (26, 27). The current study revealed that the expression of *Itg* α3, α4, αv, β1, and β3 subunits was significantly increased in comparison with the control group in the diabetic rat model.

Zhou and colleagues illustrated that *Itg* αv, β1, and β3 subunits genes expression rose in renal cortex of diabetic nephropathy rat model (13). Similarly, Sawada and colleagues reported in the early stage of human diabetic nephropathy, α3 and β1 genes expression increased (14). From our point of view, effects of metabolic disorders such as DM probably highlight the reasons and causes of unexplained infertility. In female reproductive system, hormonal changes during the proliferative phase and secretory phase leads to the presence of *Itg* family molecules in different levels (5, 28). For this reason, the exact time for evaluation of gene expression in the endometrium has high importance. Our study reveals that DM could affect *Itg* genes expression at the time of implantation window. It means the expression of *Itg* α3, α4, αv, β1, and β3 subunits in diabetic rat models has increased significantly in comparison with the control group. Previously, the dysregulation of *Itg* genes expression subunits has been accepted as a reason for the reduction in conception rate and receptivity of the endometrium. Also, it could decrease the chance of embryo adhesion to the endometrial epithelium (29). According to the literature, increasing blood glucose levels could modulate the synthesis and function of *Itg* in various tissues which by self can lead to the disorganized cell-to-cell- or cell-to-matrix adhesion. These molecular disarrangements could change the natural behavior of these tissues and cells (30). Therefore, DM, as a metabolic disease, deeply impress on the structural status of all of the tissues. Due to the high expression of *Itg* subunits gene in DM and the possibility of increased adhesion of endometrial cells to each other and extracellular matrix, it seems the invasion of the blastocyst into endometrial epithelium could be reduced and the implantation rate probably falls down (24).

In this study, two common oral hypoglycemic drugs, that is, metformin and pioglitazone were administrated in diabetic rats. Our results reveal that pioglitazone had a better effect on the reduction of all *Itg* subunit genes expression due to its impression on genes expression pathway, which previously rose in the diabetic group, while metformin reduced the level of αv, β1, and β3 gene expression but elevated the values of α3 and α4 subunit genes. From our point of view, these different effects between two drugs are due to their functional mechanisms. The family of TZDs drug, such as pioglitazone, attaches to PPARγ. Pioglitazone is a “highly selective” agonist for PPARγ. The activation of this receptor activates several DNA transcription factors that are involved in several biological and metabolic pathways such as differentiation, atherosclerosis, adipogenesis, and insulin sensitivity (31). That is why, nowadays, pioglitazone attract more attention and clinical interest. These results indicate that pioglitazone (as a member of the TZDs family) has a more effective impression on controlling the expression of *Itg* genes. Which may be due to interaction with PPARγ (a key regulator of some genes expression) and effect on DNA transcription in endometrial tissue (24).

## 5. Conclusion

DM significantly increased the expression of all the studied *Itg* subunits, therefore untreated diabetes could be potentially considered as one of the elements in embryo implantation failure. Our study suggests that if no clinical contraindication exists and after evaluation of the *Itg* proteins expression, pioglitazone could be assumed as the elective drug choice to control the upregulation of endometrial *Itg* in diabetic rat models at the time of implantation.

##  Conflict of Interest

The authors declare that there is no conflict of interest.
